# Association of social network size and composition with physical activity in Korean middle-aged adults

**DOI:** 10.4178/epih.e2020070

**Published:** 2020-11-25

**Authors:** Moon Su Kwak, So Mi Jemma Cho, Jee-Seon Shim, Dae Jung Kim, Yoosik Youm, Hyeon Chang Kim

**Affiliations:** 1 Yonsei University College of Medicine, Seoul, Korea; 2 Department of Preventive Medicine, Yonsei University College of Medicine, Seoul, Korea; 3 Cardiovascular and Metabolic Diseases Etiology Research Center, Yonsei University College of Medicine, Seoul, Korea; 4 Integrative Research Center for Cerebrovascular and Cardiovascular Diseases, Yonsei University College of Medicine, Seoul, Korea; 5 Department of Endocrinology and Metabolism, Ajou University School of Medicine, Suwon, Korea; 6 Department of Sociology, Yonsei University, Seoul, Korea

**Keywords:** Social network, Lifestyle modification, Physical activity

## Abstract

**OBJECTIVES:**

Physical activity (PA) is an established protective factor for many chronic diseases. Numerous studies have established positive relationships between social networks and PA. Accordingly, this study examined the relationship between social network structures (specifically the network size and the number and proportion of same-sex alters) and self-reported PA in Korean middle-age adults, where the term “alter” refers to a respondent’s social network members.

**METHODS:**

We analyzed 8,092 participants of the Cardiovascular and Metabolic Diseases Etiology Research Center cohort. We assessed the association between each network structure variable and PA level using a linear regression model. Then, we employed logistic regression to evaluate associations between social network structure and adherence to guideline-recommended exercise levels. Socio-demographic factors and health status measures were used as covariates.

**RESULTS:**

In both sexes, the social network size and proportion of same-sex network members showed positive relationships with total and moderate-to-vigorous PA. Notably, female participants with a greater number of kin were more likely to satisfy the recommended amount of total PA.

**CONCLUSIONS:**

These findings suggest that large scale, same-sex intervention programs can help to achieve recommended PA regimens.

## INTRODUCTION

Physical activity (PA) is defined as any bodily movement that results in energy expenditure [1]. Many researchers have studied the protective relationship of PA with mental [2,3], cardiovascular [4,5], and metabolic [6] health. As an important lifestyle factor for a wide array of diseases, PA can be promoted by social relationships. Some studies have shown that large network size, frequent contact with network members, and acquaintance with demographically homogeneous network members were associated with higher PA levels [7,8]. Many have speculated that greater exposure to information regarding the benefits of PA and participatory opportunities contribute to such findings. Others have proposed that social interactions boost self-esteem, and thereby increase motivation to care for one’s health [7-9]. Specifically, social network promotes PA by providing social support, role modeling, behavioral guidance, and/or required tools/infrastructure [10,11].

As the protective effects of PA have been repeatedly emphasized, it is crucial to reduce physical inactivity at a population level. Previous studies [12,13] have investigated the relationship between social network and PA in Korean elderly and adolescent populations; however, no study has focused solely on middle-aged population. Hitherto, we examined the association between social network structure and PA level in community-dwelling, middle-aged, Korean adults. Specifically, we explored whether the social network size and composition were associated with total and moderate-to-vigorous physical activity (MVPA).

## MATERIALS AND METHODS

### Study participants

The Cardiovascular and Metabolic Diseases Etiology Research Center (CMERC) cohort consists of two distinct community-dwelling and high-risk clinic populations. In this study, we focused on community-dwelling adults due to the known heterogeneity of social networks in the patient population. Between 2013 and 2018, 8,097 adults (2,808 male, 5,289 female) underwent baseline examination including blood tests and questionnaires on demographics, disease history, health behavior, and psychosocial factors. Detailed descriptions of the CMERC study can be found elsewhere [14]. After excluding 5 participants with extremely high PA levels a total of 8,092 participants were included in the final analysis.

### Physical activity assessment

PA was assessed using the International Physical Activity Questionnaire-Short Form [15]. We obtained combined functional and leisure-time PA, which was described in metabolic equivalent of task (MET) in minutes per week. Based on the previous literature [16], we applied weightings of 3.3, 4.0, and 8.0 METs for light-intensity, moderate-intensity, and vigorous-intensity activities, respectively, to calculate total and MVPA METs. We, then, categorized the participants into MET quartiles accordingly. Examples of light activities included leisurely walking, moderate activities included cycling, and vigorous activities included fast-paced running or strenuous anaerobic training.

### Social network structure assessment

The CMERC cohort study utilized a social network module called *name generator*, which was originally developed for the General Social Survey in the United States to study social processes with respect to the government system and interpersonal relationships [17]. The Korean version of the *name generator* has previously been implemented in nationwide social surveys and has been demonstrated to coherently capture important network properties in the context of health studies [18]. In this study, we utilized the *name generator* module to characterize egocentric social networks.

In detail, participants were asked to list their spouse (if any) and acquaintances (up to 5) with whom they had discussed important matters over the last 12 months. Respondents were referred to as “ego” and their social network members as “alters.” Important matters included good or bad experiences, problems, or concerns that the ego had. Next, participants were asked to characterize their relationship with each network member as follows: spouse, parent, child, sibling, neighbor, friend, religious affiliates, health professional, social worker, or others. Parents, children, siblings, and other relatives including in-laws were classified as kin. Then, we inquired on the network members’ sex and cohabitation status. Next, participants were asked to rate their emotional closeness to each network member from “not very close” to “extremely close” (coded 1 to 4, respectively). We, then, averaged the scores to obtain a mean closeness score to alters. Participants were also asked to report the conversation frequency with each alter on an 8-point scale, ranging from “less than once per year” to “every day.” Scores were assigned according to the approximate number of days per year (e.g., “every day”=365; “once a month”=12) and were summed across all alters to obtain a measure of overall volume of contact.

From the module, we synthesized measures of social network size (0-1, 2, 3, ≥ 4) and the number (0,1, 2, 3, ≥ 4) and proportion (0-25, 25-50, 50-75, 75-100%) of same-sex alters and kin. The difference between people with 0 network size and those with 1 network size 1, and the results of the analysis separating 0 and 1 are presented in Supplementary Materials 1-3.

### Covariates

We collected demographics, disease history, and health behaviors from face-to-face interviews using structured questionnaires. Participants were classified as current, former, or non-drinkers. Obesity was defined based on the 2018 Korean Society for the Study of Obesity guideline [19] as a body mass index ≥ 25.0 kg/m^2^. Hypertension was defined based on the 2018 Korean Society of Hypertension guideline [20] for high blood pressure as a systolic blood pressure ≥ 140 mmHg, diastolic blood pressure ≥ 90 mmHg, or taking antihypertensive medications. Diabetes mellitus was defined based on the 2019 Korean Diabetes Association guideline [21] as an eight-hour fasting glucose of ≥ 126 mg/dL, hemoglobin A1c level ≥ 6.5%, taking glucose-lowering agent, or receiving insulin injections. Hypercholesterolemia and hypertriglyceridemia were defined based on the 2018 Korean Society of Lipid and Atherosclerosis guideline [22] as a total cholesterol ≥ 240 mg/dL, a triglyceride level ≥ 200 mg/dL, or taking lipid-lowering drugs. All bioassays were performed at a single designated research laboratory (Seoul Clinical Laboratory R&D Center, Seoul, Korea).

### Statistical analysis

We compared the baseline characteristics by sex using independent t-test. Next, we evaluated the linear associations between network structure variables and PA level, adjusting for age, body mass index, marital status, cohabitation status, education level, household income, occupation, smoking, and alcohol drinking. Furthermore, we assessed categorical association between social network structure and adherence to guideline-recommended [23] exercise engagement by multivariable logistic regression. Participants who achieved 500 MET-min/wk of total PA or 150 min/wk of MVPA were classified as guideline-adherent.

All statistical tests were 2-sided, and statistical significance was set at a p-value < 0.05. All analyses were performed using SAS version 9.4 (SAS Institute Inc., Cary, NC, USA).

### Ethics statement

The CMERC cohort study was approved by the institutional review boards of Severance Hospital, Yonsei University Health System, Seoul, Korea (4-2013-0661) and Ajou University School of Medicine, Suwon, Korea (AJIRB-BMR-SUR-13-272).

We obtained written informed consent from all participants prior to the baseline examination.

## RESULTS

Table 1 presents the general characteristics of study participants by sex. On average, females were older than males (51.8 vs. 50.7 years). Although males were more likely to be more obese (48.2 vs. 18.0%), they also performed more PA than females (2,425.0 vs. 1846.8 total MET-min/wk; 243.5 vs. 150.8 MVPA MET-min/wk).

Table 2 shows linear association between social network structure and PA level. In both sexes, the network size was positively associated with both total (male: β=367.2, p<0.001 ; female: β=271.3, p<0.001) and MVPA (male: β=30.2, p<0.001; female: β=20.5, p<0.001) METs. The number of same-sex alters also showed positive relationships with both total (male: β=311.2, p<0.001; female: β=230.6, p<0.001) and MVPA (male: β=26.6, p<0.001; female: β=17.3, p<0.001) METs. The associations remained robust for the proportion of same-sex alters. Next, the number of kin showed positive relationship with total PA (male: β=206.8, p=0.002; female: β=92.7, p=0.004) and MVPA (male: β=24.0, p=0.011; female: β=10.4, p=0.014) in both sexes. However, in males, the proportion of kin showed a negative relationship with total PA (β=-155.2, p=0.004) yet no significant relationship with MVPA (β=-6.1, p=0.428). No significant associations were observed with the proportion of kin in female.

Table 3 shows the associations between social network structure and adherence to the guideline-recommended exercise levels. Male participants with a larger network size and a higher number and proportion of same-sex alters were more likely to engage in the recommended amount of PA. However, no meaningful associations were observed based on the number or proportion of kin within their networks. Likewise, female participants with a larger network size and a higher proportion of same-sex alters were more likely to achieve the recommended amount of PA. Furthermore, those with greater number of kin within the network were also likely to engage in the recommended amount of total PA, but not MVPA.

## DISCUSSION

In this study, we found sex-specific associations of social network size and the proportion of same-sex alters and kin with PA levels. Unlike male, female with a greater number of kin were more likely to satisfy the recommended amount of PA in terms of total PA. In both sexes, all social network composition variables, except for the proportion of kin, showed positive relationships with PA of all rigors.

Our findings support and complement previously reported associations between social networks and PA. The 1990 Ontario Health Survey showed positive associations between social quantity (number of close relatives and friends) and self-reported PA [24]. In another study [25] based on residents of 12 low-income housing communities in metropolitan Boston, residents with smaller social networks were significantly less physically active based on pedometer measurements than their counterparts with larger social networks. One proposed mechanism is that a large network size enables individuals to receive more information on the benefits of PA and opportunities to participate [8]. Others speculate that a larger network can elevate one’s self esteem by positive interactions within the network, thereby increasing motivation to attend to one’s health [7].

In addition, our results indicate that the number and proportion of same-sex members within one’s social network were positively associated with PA level in both sexes. In a study based on 6 Catholic schools in Calgary [26], social density and friendship intimacy were positively associated with meeting the recommended amount of MVPA (self-reported). The authors observed that for male, the presence of physically active friends within the network encouraged ego to engage in more PA, while in female, emotional support from female friends inspired greater PA [26]. In a Brazilian occupational study [27], material support was positively associated with PA in female (odds ratio, 2.76). Affective support was associated with time spent on leisure-time PA only in male (odds ratio, 1.80) [27].

Whereas the number of kin showed a positive association with PA levels in female, contrasting results were observed in male. Previous studies suggested that a lack of confidence on initiating PA, lack of network members who are familiar and competent with PA, negative self-perceptions regarding body image, and insufficient time for PA due to caregiving responsibilities are potential barriers to PA engagement in middle-aged male [28]. Therefore, the disproportionate family-caretaking role in Korean households [29-31] may explain the negative relationship between PA level and number of kin observed in our male participants.

Regarding the positive association between PA level and number of kin in female, family members’ motivation may encourage female to engage in PA. Indeed, previous studies [32-34] have shown that female tend to participate in PA with their family members, whereas male prefer to do so independently or with non-family members. This may furnish a natural explanation of higher PA in female participants with a greater number of kin.

This study has several strengths. To our knowledge, few studies have examined the associations between social network structure and PA in middle-aged Korean adults. Considering that lifetime exposure to PA is crucial in the context of primary and secondary prevention of chronic diseases, our study may provide evidence for more holistic and persistent intervention programs. Second, this study included a large number of participants, thereby yielding statistically robust results. Third, we investigated various aspects of the social network composition, which enabled us to specify characteristics of the network structure associated with PA.

However, there are some limitations to be considered. First, as the study design was cross-sectional, we were unable to draw conclusions regarding the causality of associations between social network structure and PA. Second, we relied on a subjective measure of PA, which can yield differential misclassification bias. Lastly, the associations between social network structure and MVPA were inconsistent. Future studies based on objective measures of MVPA that can effectively distinguish between functional and leisure-time PAs may further strengthen the findings.

In conclusion, we observed sex-specific associations of social network size and network composition (the proportion of same-sex alters) with PA. Beyond individual-level characteristics, a consideration of interpersonal aspects may be helpful to achieve optimal PA.

## Figures and Tables

**Table 1.** General characteristics of the study population (n=8,092)

Variables	Male (n=2,805)	Female (n=5,287)	p-value^1^
Age (yr)	50.74±9.34	51.78±8.32	<0.001
Marital status			
Married and cohabiting	2,596 (92.5)	4,457 (84.3)	<0.001
Married yet not cohabiting	13 (0.5)	63 (1.2)	
Widowed	8 (0.3)	300 (5.7)	
Divorced	43 (1.5)	287 (5.4)	
Unmarried	145 (5.2)	180 (3.4)	
Cohabitation			
With partner or family member	2,720 (97.0)	5,014 (94.8)	<0.001
With non-family member	14 (0.5)	20 (0.4)	
Single	71 (2.5)	253 (4.8)	
Education level			
Elementary school or below	78 (2.8)	339 (6.4)	<0.001
Middle school	160 (5.7)	620 (11.7)	
High school	994 (35.4)	2,506 (47.4)	
College/university or above	1,573 (56.1)	1,821 (34.4)	
Household income			
Low	538 (19.2)	1,369 (25.9)	<0.001
Middle-low	680 (24.2)	1,311 (24.8)	
Middle-high	758 (27.0)	1,311 (24.8)	
High	829 (29.5)	1,296 (24.5)	
Occupation			
White-collar	1,469 (52.4)	1,307 (24.7)	<0.001
Blue-collar	1,082 (38.6)	1,599 (30.2)	
Unemployed	254 (9.1)	2,381 (45.0)	
Cigarette smoking			
Non-smoker	627 (22.3)	4,973 (94.1)	<0.001
Former smoker	1,257 (44.8)	177 (3.3)	
Current smoker	921 (32.8)	137 (2.6)	
Alcohol consumption			
Non-drinkers	272 (9.7)	1,769 (33.5)	<0.001
Former drinkers	158 (5.6)	144 (2.7)	
Current drinkers	2,375 (84.7)	3,374 (63.8)	
Physical activity			
Total MET (MET min/wk)	2,425.0±3,371.4	1,846.8±2,594.2	
MVPA (MET min/wk)	243.5±483.8	150.8±343.3	<0.000
Sedentary time (min/wk)	407.9±208.8	341.8±180.4	<0.001
Morbidities			
Obese	1,353 (48.2)	1,456 (27.5)	<0.001
Hypertension	624 (22.2)	813 (15.4)	<0.001
Diabetes mellitus	383 (13.6)	403 (7.6)	<0.001
Dyslipidemia	384 (13.7)	904 (17.1)	<0.001

Values are presented as mean ± standard deviation or number (%). MET, metabolic equivalent of task; MVPA, moderate-to-vigorous physical activity.

1 From the independent t-test.

**Table 2.** Associations between social network structure and physical activity level (n=8,092)

Characteristics	Total METs				MVPA			
	Unadjusted		Adjusted^1^		Unadjusted		Adjusted^1^	
	β	p-value	β	p-value	β	p-value	β	p-value
Male (n=2,805)								
Network size	315.2	<0.001	367.2	<0.001	24.1	0.001	30.2	<0.001
No. of same-sex alters	272.6	<0.001	311.2	<0.001	22.6	0.000	26.6	<0.001
Proportion of same-sex alters	314.1	<0.001	360.0	<0.001	27.9	0.001	31.5	<0.001
No. of kin	173.3	0.008	206.8	0.002	17.7	0.058	24.0	0.011
Proportion of kin	-142.7	0.007	-155.2	0.004	-6.5	0.396	-6.1	0.428
Female (n=5,287)								
Network size	236.1	<0.001	271.3	<0.001	18.1	<0.001	20.5	<0.001
No. of same-sex alters	209.7	<0.001	230.6	<0.001	15.5	<0.001	17.3	<0.001
Proportion of same-sex alters	245.3	<0.001	274.8	<0.001	16.1	0.001	19.2	<0.001
No. of kin	69.4	0.027	92.7	0.004	9.3	0.025	10.4	0.014
Proportion of kin	-68.2	0.029	-71.2	0.024	-1.0	0.810	-1.9	0.649

MET, metabolic equivalent of task; MVPA, moderate-to-vigorous physical activity.

1 Adjusted for age, body mass index, marital status, cohabitation status, education level, household income, occupation, smoking, alcohol drinking, and obesity.

**Table 3.** Associations between social network structure and adherence to guideline-recommended PA in sexes

Social network structure			No. of participants	Total METs			MVPA		
				Meeting the recommended PA level, n (%)	Unadjusted OR (95% CI)	Adjusted OR (95% CI)^1^	Meeting the recommended PA level, n (%)	Unadjusted OR (95% CI)	Adjusted OR (95% CI)^1^
Male (n=2,805)									
	Network size								
		0-1	665	427 (64.2)	1.00 (reference)	1.00 (reference)	231 (34.7)	1.00 (reference)	1.00 (reference)
		2	407	287 (70.5)	1.33 (1.02, 1.74)	1.41 (1.08, 1.85)	150 (36.9)	1.10 (0.85, 1.42)	1.14 (0.88, 1.48)
		3	601	440 (73.2)	1.52 (1.20, 1.94)	1.61 (1.26, 2.06)	218 (36.3)	1.07 (0.85, 1.35)	1.12 (0.88, 1.41)
		≥4	1,132	917 (81.0)	2.38 (1.91, 2.95)	2.60 (2.07, 3.26)	517 (45.7)	1.58 (1.30, 1.93)	1.66 (1.36, 2.04)
	No. of same-sex alters								
		0	723	467 (64.6)	1.00 (reference)	1.00 (reference)	250 (34.6)	1.00 (reference)	1.00 (reference)
		1	523	374 (71.5)	1.38 (1.08, 1.76)	1.41 (1.10, 1.81)	188 (36.0)	1.06 (0.84, 1.34)	1.09 (0.86, 1.38)
		2	603	459 (76.1)	1.75 (1.37, 2.22)	1.83 (1.43, 2.34)	238 (39.5)	1.23 (0.99, 1.54)	1.28 (1.02, 1.61)
		3	370	287 (77.6)	1.90 (1.42, 2.53)	2.04 (1.52, 2.74)	168 (45.4)	1.57 (1.22, 2.03)	1.64 (1.26, 2.13)
		≥4	586	484 (82.6)	2.60 (2.00, 3.38)	2.77 (2.12, 3.63)	272 (46.4)	1.64 (1.31, 2.05)	1.70 (1.35, 2.14)
	Percent of same-sex alters								
		0-25	765	499 (65.2)	1.00 (reference)	1.00 (reference)	270 (35.3)	1.00 (reference)	1.00 (reference)
		25-50	665	499 (75.0)	1.60 (1.27, 2.02)	1.65 (1.30, 2.09)	254 (38.2)	1.13 (0.91, 1.41)	1.16 (0.93, 1.45)
		50-75	863	652 (75.6)	1.65 (1.33, 2.04)	1.74 (1.39, 2.17)	354 (41.0)	1.28 (1.04, 1.56)	1.31 (1.07, 1.61)
		75-100	512	421 (82.2)	2.47 (1.88, 3.23)	2.54 (1.92, 3.36)	238 (46.5)	1.60 (1.27, 2.00)	1.63 (1.29, 2.06)
	No. of kin								
		0	503	360 (71.5)	1.00 (reference)	1.00 (reference)	188 (37.4)	1.00 (reference)	1.00 (reference)
		1	1,604	1154 (72.0)	1.02 (0.82, 1.27)	1.08 (0.85, 1.36)	625 (22.3)	1.07 (0.87, 1.32)	1.11 (0.89, 1.38)
		2	360	291 (80.8)	1.68 (1.21, 2.32)	1.80 (1.29, 2.51)	155 (43.1)	1.27 (0.96, 1.67)	1.32 (1.00, 1.75)
		3	212	163 (76.9)	1.32 (0.91, 1.92)	1.41 (0.96, 2.06)	88 (41.5)	1.19 (0.86, 1.65)	1.25 (0.90, 1.75)
		≥4	126	103 (81.75)	1.78 (1.09, 2.91)	2.04 (1,23, 3.37)	60 (47.6)	1.52 (1.03, 2.26)	1.64(1.10, 2.46)
	Percent of kin								
		0-25	1,016	772 (76.0)	1.00 (reference)	1.00 (reference)	425 (41.8)	1.00 (reference)	1.00 (reference)
		25-50	855	644 (75.3)	0.97 (0.78, 1.19)	1.00 (0.80, 1.23)	330 (38.6)	0.87 (0.73, 1.05)	0.89 (0.74, 1.08)
		50-75	191	156 (81.7)	1.41 (0.95, 2.09)	1.40 (0.94, 2.10)	77 (40.3)	0.94 (0.69, 1.29)	0.94 (0.68, 1.29)
		75-100	743	499 (67.2)	0.65 (0.52, 0.80)	0.64 (0.52, 0.80)	284 (38.2)	0.86 (0.71, 1.04)	0.87 (0.71, 1.05)
Female (n=5,287)									
	Network size								
		0-1	628	373 (59.4)	1.00 (reference)	1.00 (reference)	167 (26.6)	1.00 (reference)	1.00 (reference)
		2	850	526 (61.9)	1.11 (0.90, 1.37)	1.12 (0.90, 1.38)	219 (25.8)	0.96 (0.76, 1.21)	0.96 (0.76, 1.21)
		3	1,319	896 (67.9)	1.45 (1.19, 1.76)	1.52 (1.24, 1.86)	363 (27.5)	1.05 (0.85, 1.30)	1.05 (0.85, 1.30)
		≥4	2,490	1,803 (72.4)	1.79 (1.50, 2.15)	1.94 (1.61, 2.34)	806 (32.4)	1.32 (1.09, 1.61)	1.32 (1.09, 1.61)
	No. of same-sex alters								
		0	645	389 (60.3)	1.00 (reference)	1.00 (reference)	177 (27.4)	1.00 (reference)	1.00 (reference)
		1	909	549 (60.4)	1.00 (0.82, 1.23)	1.02 (0.83, 1.26)	225 (24.8)	0.87 (0.69, 1.09)	0.87 (0.69, 1.10)
		2	1,349	931 (69.0)	1.47 (1.21, 1.78)	1.54 (1.26, 1.88)	392 (29.1)	1.08 (0.88, 1.34)	1.11 (0.89, 1.37)
		3	953	665 (69.8)	1.52 (1.23, 1.87)	1.62 (1.31, 2.00)	268 (28.1)	1.03 (0.83, 1.29)	1.07 (0.85, 1.34)
		≥4	1,431	1,064 (74.4)	1.91 (1.57, 2.32)	2.05 (1.68, 2.51)	493 (34.5)	1.39 (1.13, 1.71)	1.45 (1.18, 1.78)
	Percent of same-sex alters								
		0-25	679	409 (60.2)	1.00 (reference)	1.00 (reference)	182 (26.8)	1.00 (reference)	1.00 (reference)
		25-50	1,053	667 (63.3)	1.14 (0.94, 1.39)	1.16 (0.95, 1.42)	284 (27.0)	1.01 (0.81, 1.25)	1.00 (0.80, 1.25)
		50-75	1,988	1,380 (69.4)	1.50 (1.25, 1.80)	1.56 (1.30, 1.88)	578 (29.1)	1.12 (0.92, 1.36)	1.12 (0.91,1.36)
		75-100	1,567	1,142 (72.9)	1.77 (1.47, 2.15)	1.94 (1.59, 2.36)	511 (32.6)	1.32 (1.08, 1.61)	1.45 (1.18,1.78)
	No. of kin								
		0	717	461 (64.3)	1.00 (reference)	1.00 (reference)	186 (25.9)	1.00 (reference)	1.00 (reference)
		1	2,129	1447 (68.0)	1.18 (0.99, 1.41)	1.25 (1.03, 1.51)	660 (31.0)	1.28 (1.06, 1.55)	1.26 (1.03, 1.54)
		2	1,251	852 (68.1)	1.19 (0.98, 1.44)	1.27 (1.03, 1.55)	353 (28.2)	1.12 (0.91, 1.38)	1.10 (0.88, 1.37)
		3	736	511 (69.4)	1.26 (1.01, 1.57)	1.36 (1.08, 1.70)	214 (29.1)	1.17 (0.93, 1.47)	1.17 (0.92, 1.48)
		≥4	454	327 (72.0)	1.43 (1.11, 1.85)	1.53 (1.17, 2.00)	142 (31.3)	1.30 (1.00, 1.68)	1.27 (0.97, 1.66)
	Percent of kin								
		0-25	1,442	988 (68.5)	1.00 (reference)	1.00 (reference)	438 (30.4)	1.00 (reference)	1.00 (reference)
		25-50	1,697	1,182 (69.65)	1.06 (0.91, 1.23)	1.07 (0.91, 1.25)	506 (29.8)	0.97 (0.84, 1.13)	0.95 (0.81, 1.11)
		50-75	766	536 (70.0)	1.07 (0.89, 1.30)	1.08 (0.89, 1.31)	227 (29.6)	0.97 (0.80, 1.17)	0.93 (0.77, 1.13)
		75-100	1,382	892 (64.5)	0.84 (0.72, 0.98)	0.82 (0.69, 0.96)	384 (27.8)	0.88 (0.75, 1.04)	0.84 (0.71, 0.99)

PA, physical activity; MET, metabolic equivalent of task; MVPA, moderate-to-vigorous physical activity; OR, odds ratio; CI, confidence interval.

1 Adjusted for age, body mass index, marital status, cohabitation status, education level, household income, occupation, smoking, alcohol drinking, and obesity.
