# Supplementary material for: Association of social network size and composition with physical activity in Korean middle-aged adults
Source: Epidemiol Health. 2020 Nov 25;42:e2020070. doi: 10.4178/epih.e2020070 (PMC8137373; doi:10.4178/epih.e2020070)
Supplement: Supplementary Material 1. [file epih-42-e2020070-suppl1.docx]

**Supplementary Materials**

| Supplementary Material 1. General characteristics of people with network size 0 and 1 | | | | | | | | | | | |
| --- | --- | --- | --- | --- | --- | --- | --- | --- | --- | --- | --- |
| Variable | Male | | | | |  | Female | | | | |
|  | Network size 0 (n=16) | |  | Network size 1 (n=649) | |  | Network size 0 (n=40) | |  | Network size 1 (n=588) | |
| Age, year | 47.3 |  |  | 53.4 |  |  | 55.4 |  |  | 53.5 |  |
| Marital status |  |  |  |  |  |  |  |  |  |  |  |
| Married and cohabiting | 0 | 0% |  | 622 | 95.8% |  | 0 | 0% |  | 482 | 82.0% |
| Married yet not cohabiting | 0 | 0% |  | 5 | 0.8% |  | 0 | 0% |  | 4 | 0.7% |
| Widowed | 0 | 0% |  | 4 | 0.6% |  | 20 | 50.0% |  | 35 | 6.0% |
| Divorced | 8 | 50% |  | 4 | 0.6% |  | 13 | 32.5% |  | 47 | 8.0% |
| Unmarried | 8 | 50% |  | 14 | 2.2% |  | 7 | 17.5% |  | 20 | 3.4% |
| Cohabitation |  |  |  |  |  |  |  |  |  |  |  |
| With partner or family member | 10 | 62.5% |  | 638 | 98.3% |  | 26 | 65.0% |  | 551 | 93.7% |
| With non-family member | 2 | 12.5% |  | 3 | 0.5% |  | 1 | 2.5% |  | 1 | 0.2% |
| Single | 4 | 25.0% |  | 8 | 1.2% |  | 13 | 32.5% |  | 36 | 6.1% |
| Education level |  |  |  |  |  |  |  |  |  |  |  |
| Elementary school or below | 3 | 18.8% |  | 34 | 5.2% |  | 2 | 5.0% |  | 63 | 10.7% |
| Middle school | 1 | 6.3% |  | 58 | 8.9% |  | 12 | 30.0% |  | 112 | 19.1% |
| High school | 7 | 43.8% |  | 261 | 40.2% |  | 19 | 47.5% |  | 266 | 45.3% |
| College/university or above | 5 | 31.3% |  | 296 | 45.6% |  | 7 | 17.5% |  | 146 | 24.9% |
| Household income |  |  |  |  |  |  |  |  |  |  |  |
| Low | 9 | 56.3% |  | 138 | 21.3% |  | 24 | 60.0% |  | 163 | 27.7% |
| Middle-low | 1 | 6.3% |  | 146 | 22.5% |  | 8 | 20.0% |  | 134 | 22.8% |
| Middle-high | 3 | 18.8% |  | 189 | 29.1% |  | 5 | 12.5% |  | 144 | 24.5% |
| High | 3 | 18.8% |  | 176 | 27.1% |  | 3 | 7.5% |  | 147 | 25.0% |
| Occupation |  |  |  |  |  |  |  |  |  |  |  |
| White collar | 5 | 31.3% |  | 78 | 12.0% |  | 14 | 35.0% |  | 245 | 41.7% |
| Blue collar | 7 | 43.8% |  | 292 | 45.0% |  | 18 | 45.0% |  | 226 | 38.4% |
| Unemployed | 4 | 25.0% |  | 279 | 43.0% |  | 8 | 20.0% |  | 117 | 19.9% |
| Cigarette smoking |  |  |  |  |  |  |  |  |  |  |  |
| Non-smoker | 5 | 31.3% |  | 137 | 21.1% |  | 32 | 80.0% |  | 550 | 93.5% |
| Former smoker | 6 | 37.5% |  | 318 | 49.0% |  | 6 | 15.0% |  | 23 | 3.9% |
| Current smoker | 5 | 31.3% |  | 194 | 29.9% |  | 2 | 5.0% |  | 15 | 2.6% |
| Alcohol consumption |  |  |  |  |  |  |  |  |  |  |  |
| Non-drinker | 2 | 12.5% |  | 86 | 13.3% |  | 18 | 45.0% |  | 223 | 37.9% |
| Former drinker | 1 | 6.3% |  | 35 | 5.4% |  | 2 | 5.0% |  | 16 | 2.7% |
| Current drinker | 13 | 81.3% |  | 528 | 81.4% |  | 20 | 50.0% |  | 349 | 59.4% |
| Physical activity |  |  |  |  |  |  |  |  |  |  |  |
| Total MET-min/week | 3574.9 |  |  | 1882.9 |  |  | 1644.8 |  |  | 1404.6 |  |
| MVPA-min/week | 468.8 |  |  | 199.2 |  |  | 116.3 |  |  | 125.9 |  |
| Sedentary time-min/week | 370 |  |  | 394.2 |  |  | 380.3 |  |  | 326.6 |  |
| Morbidity |  |  |  |  |  |  |  |  |  |  |  |
| Obese | 10 | 37.5% |  | 307 | 47.3% |  | 12 | 30.0% |  | 174 | 29.6% |
| Hypertension | 3 | 18.8% |  | 176 | 27.1% |  | 8 | 20.0% |  | 88 | 15.0% |
| Diabetes mellitus | 3 | 18.8% |  | 116 | 17.9% |  | 5 | 12.5% |  | 46 | 7.8% |
| Dyslipidemia | 2 | 12.5% |  | 106 | 16.3% |  | 7 | 17.5% |  | 109 | 18.5% |
| MET, metabolic equivalent of task; MVPA, moderate-to-vigorous physical activity  Values are presented as mean ± standard deviation or number (%).  *P*-value was derived from the independent t-test. | | | | | | | | | | | |
