# Supplementary material for: Association of social network size and composition with physical activity in Korean middle-aged adults
Source: Epidemiol Health. 2020 Nov 25;42:e2020070. doi: 10.4178/epih.e2020070 (PMC8137373; doi:10.4178/epih.e2020070)
Supplement: Supplementary Material 2. [file epih-42-e2020070-suppl2.docx]

| **Supplementary Material 2.** Association between social network size and physical activity level (network size 0 and 1 analyzed separately) | | | | | | | | | | | |
| --- | --- | --- | --- | --- | --- | --- | --- | --- | --- | --- | --- |
| Social network size | Total MET | | | | |  | MVPA | | | | |
|  | Unadjusted | |  | Adjusted | |  | Unadjusted | |  | Adjusted | |
|  | β | *p*-value |  | β | *p*-value |  | β | *p*-value |  | β | *p*-value |
| Male (n=2,805) |  |  |  |  |  |  |  |  |  |  |  |
| Network size | 305.2 | <0.0001 |  | 358.9 | <0.0001 |  | 22.8 | 0.0024 |  | 29.3 | 0.0001 |
| Female (n=5,287) |  |  |  |  |  |  |  |  |  |  |  |
| Network size | 229.4 | <0.0001 |  | 265.2 | <0.0001 |  | 17.7 | <0.0001 |  | 20.1 | <0.0001 |
| MET, metabolic equivalent of task; MVPA, moderate-to-vigorous physical activity  Adjusted for age, body mass index, marital status, cohabitation, education level, household income, occupation, smoking, alcohol drinking, and obesity. | | | | | | | | | | | |
