# Supplementary material for: Association of social network size and composition with physical activity in Korean middle-aged adults
Source: Epidemiol Health. 2020 Nov 25;42:e2020070. doi: 10.4178/epih.e2020070 (PMC8137373; doi:10.4178/epih.e2020070)
Supplement: Supplementary Material 3. [file epih-42-e2020070-suppl3.docx]

| **Supplementary Material 3.** Association between social network size and adherence to guideline-recommended physical activity (network size 0 and 1 analyzed separately) | | | | | | | | | |
| --- | --- | --- | --- | --- | --- | --- | --- | --- | --- |
| Social network size | No. of participant | Total MET | | |  | MVPA | | | |
|  |  | Meeting the recommended PA level, n (%) | Unadjusted OR (95% CI) | Adjusted OR^*^ (95% CI) |  | Meeting the recommended PA level, n (%) | Unadjusted OR (95% CI) | Adjusted OR^*^(95% CI) |  |
| Male (n=2,805) | |  |  |  |  |  |  |  |  |
| 0 | 16 | 12 (75.0) | 1.00 (Ref) | 1.00 (Ref) |  | 5 (31.3) | 1.00 (Ref) | 1.00 (Ref) |  |
| 1 | 649 | 415 (63.9) | 0.59 (0.19, 1.85) | 0.74 (0.22, 2.47) |  | 226 (34.8) | 1.18 (0.40, 3.42) | 1.39 (0.46, 4.23) |  |
| 2 | 407 | 287 (70.5) | 0.80 (0.25, 2.52) | 1.05 (0.31, 3.52) |  | 150 (36.9) | 1.28 (0.44, 3.77) | 1.58 (0.52, 4.83) |  |
| 3 | 601 | 440 (73.2) | 0.91 (0.29, 2.87) | 1.20 (0.36, 4.01) |  | 218 (36.3) | 1.25 (0.43, 3.65) | 1.54 (0.51, 4.69) |  |
| 4+ | 1132 | 917 (81.0) | 1.42 (0.45, 4.45) | 1.94 (0.58, 6.43) |  | 517 (45.7) | 1.85 (0.64, 5.36) | 2.30 (0.76, 6.94) |  |
| Female (n=5,287) | |  |  |  |  |  |  |  |  |
| 0 | 40 | 22 (55.0) | 1.00 (Ref) | 1.00 (Ref) |  | 5 (12.5) | 1.00 (Ref) | 1.00 (Ref) |  |
| 1 | 588 | 351 (59.7) | 1.21 (0.64, 2.31) | 1.37 (0.70. 2.66) |  | 162 (27.6) | 2.66 (1.03, 6.91) | 2.50 (0.95, 6.58) |  |
| 2 | 850 | 526 (61.9) | 1.33 (0.70, 2.51) | 1.50 (0.78, 2.89) |  | 219 (25.8) | 2.43 (0.94, 6.28) | 2.27 (0.86, 5.93) |  |
| 3 | 1319 | 896 (67.9) | 1.73 (0.92, 3.27) | 2.04 (1.06, 3.92) |  | 363 (27.5) | 2.66 (1.03, 6.84) | 2.50 (0.96, 6.54) |  |
| 4+ | 2,490 | 1,803 (72.4) | 2.15 (1.15, 4.03) | 2.60 (1.35, 4.99) |  | 806 (32.4) | 3.35 (1.31, 8.58) | 3.23 (1.24, 8.41) |  |
| PA, physical activity; MET, metabolic equivalent of task; MVPA, moderate-to-vigorous physical activity; OR, odds ratio; CI, confidence interval.  Adjusted for age, body mass index, marital status, cohabitation, education level, household income, occupation, smoking, alcohol drinking, and obesity. | | | | | | | | |  |
|  | | | | | | | | | |
